# Further Probing of Cu^2+^-Dependent PNAzymes Acting as Artificial RNA Restriction Enzymes

**DOI:** 10.3390/molecules24040672

**Published:** 2019-02-14

**Authors:** Olivia Luige, Merita Murtola, Alice Ghidini, Roger Strömberg

**Affiliations:** 1 Department of Biosciences and Nutrition, Karolinska Institutet, Neo, 141 83 Huddinge, Stockholm, Sweden; Olivia.Luige@ki.se (O.L.); Merita.Murtola@ki.se (M.M.); alice.ghidini@pharma.ethz.ch (A.G.); 2 Department of Chemistry, University of Turku, FI-20014 Turku, Finland; 3 Institut für Pharmazeutische Wissenschaften (IPW), Eidgenössische Technische Hochschule Vladimir-Prelog-Weg 1-5/10, 8093 Zürich, Switzerland

**Keywords:** artificial ribonuclease, peptide nucleic acid, RNA cleavage, catalysis

## Abstract

Peptide nucleic acid (PNA)-neocuproine conjugates have been shown to efficiently catalyse the cleavage of RNA target sequences in the presence of Cu^2+^ ions in a site-specific manner. These artificial enzymes are designed to force the formation of a bulge in the RNA target, the sequence of which has been shown to be key to the catalytic activity. Here, we present a further investigation into the action of Cu^2+^-dependent PNAzymes with respect to the dependence on bulge composition in 3- and 4-nucleotide bulge systems. Cu^2+^-dependent PNAzymes were shown to have a clear preference for 4-nucleotide bulges, as the cleavage of 3-nucleotide bulge-forming RNA sequences was significantly slower, which is illustrated by a shift in the half-lives from approximately 30 min to 24 h. Nonetheless, the nucleotide preferences at different positions in the bulge displayed similar trends in both systems. Moreover, the cleavage site was probed by introducing critical chemical modifications to one of the cleavage site nucleotides of the fastest cleaved 4-nucleotide RNA bulge. Namely, the exclusion of the exocyclic amine of the central adenine and the replacement of the 2′-hydroxyl nucleophile with 2′-H or 2′-OMe substituents in the RNA severely diminished the rate of RNA cleavage by the Cu^2+^-dependent PNAzyme, giving insight into the mechanism of cleavage. Moreover, the shorter recognition arm of the RNA/PNAzyme complex was modified by extending the PNAzyme by two additional nucleobases. The new PNAzyme was able to efficiently promote the cleavage of RNA when fully hybridised to a longer RNA target and even outperform the previous fastest PNAzyme. The improvement was demonstrated in cleavage studies with stoichiometric amounts of either PNAzyme present, and the extended PNAzyme was also shown to give turnover with a 10-fold excess of the RNA target.

## 1. Introduction

Selective manipulation of nucleic acid sequences is key for molecular biology research, medical analysis and the treatment of genetic diseases in the clinic. Natural nucleic acid target sequences can be affected by synthetic oligonucleotides (ONs). Moreover, the intrinsic affinity of ONs for their targets can be amplified by the inclusion of critical chemical modifications, which, in addition to improved hybridisation properties, can confer enhanced stability against degradation. Thus, synthetic ONs capable of modulating disease-related RNAs are attractive drug candidates with several approved therapies [1] and numerous clinical trials underway [2]. 

Therapeutic ONs can be designed to affect their targets by a range of different mechanisms, depending on the type of processes by which the pathogenicity of the target is manifested [2,3]. Degradation of the RNA target is one important mechanism for antisense oligonucleotides (ASOs), as the reduction of RNA levels is a viable therapeutic strategy for the treatment of many diseases. In the context of medical applications, synthetic oligonucleotides, namely gapmer ASOs and siRNAs, can trigger the catalytic cleavage of their RNA targets by recruiting endogenous enzymes RNase H or RNA-induced silencing complex (RISC), respectively [2,3]. However, their design is somewhat complicated by the need for a recognisable substrate for the corresponding enzyme, which restricts the arsenal of chemical modifications that can be used to fine tune their properties [4]. 

Site-specific cleavage of RNA target sequences could be an invaluable tool for molecular biology research, as DNA restriction enzymes that recognise short DNA target sequences and subsequently degrade them in a defined fashion are vital tools for research. An alternative way to selectively degrade RNA target sequences could be to use synthetic oligonucleotides equipped with an intrinsic ability to catalyse the cleavage of their RNA targets (artificial ribonucleases) without the assistance of native enzymes [5,6]. Oligonucleotide-based artificial nucleases (OBANs) have been shown to catalytically cleave their RNA targets in a sequence- and site-selective manner [7,8,9]. Neocuproine, as a metal chelate, has also been incorporated into a peptide nucleic acid (PNA) backbone at a central position, giving rise to PNA-based artificial ribonucleases (PNAzymes) [10,11,12,13,14]. These PNAzymes are designed to be partially complementary to the RNA target in such a way as to force the formation of an RNA bulge at the position adjacent to the chelating moiety. The single-stranded RNA bulge is then cleaved site-specifically with half-lives below 30 min in the presence of Cu^2+^ ions [12,13]. As such, PNAzymes show excellent specificity, but their further development is desirable in order to achieve even higher rates of catalytic cleavage, especially if they are to be considered for the depletion of RNA for therapeutic applications. Interestingly, the bulge sequence has been shown to have a major influence on the cleavage kinetics and the highest cleavage rates have been demonstrated with 4-nucleotide bulge sequences APyPuA-G (Py = pyrimidine, Pu = purine base), where the bulge is closed with a GT wobble base pair. A deeper understanding of the structural changes tolerated by this intricate system is critical, as it can potentially reveal even more efficiently cleaved systems, allow for modulation of the properties of PNAzymes by the conjugation of different entities if they are known not to interfere with the catalytic activity, and illustrate the specificity ensured by mismatch intolerance in the bulge or in the hybridised recognition motifs. As such, any mechanistic insights are of crucial importance for the further design of PNA-based artificial ribonucleases.

In the present report, we describe the most recent studies on our Cu^2+^-dependent PNAzymes, including investigations into RNAs forming 3-nucleotide bulges, modified RNAs with chemical modifications in the immediate vicinity of the cleavage site in the bulge, and a new extended PNAzyme designed to probe the significance of the shorter recognition arm of the RNA/PNAzyme complex. 

## 2. Results and Discussion

### 2.1. Three-Nucleotide Bulge-Forming RNA Targets

Throughout our research into sequence-specific artificial ribonucleases, the most significant improvements in cleavage rates have consistently resulted from variations of the RNA sequence in the unpaired region of the RNA/PNAzyme complex [7,8]. The three-dimensional helical structure of RNA is fascinating, as it is governed by a range of interactions, including canonical and non-canonical base-pairing via hydrogen bonding, as well as nucleobase stacking interactions, which reduce the exposure of hydrophobic surfaces to the polar environment. Structural variation within the confines of helical structures is relatively limited, but stretches of unpaired nucleotides form bulges which add to the diversity of RNA structures as architectural or recognition motifs [15]. RNA bulges are known to adopt diverse conformations, resulting from the competing interactions between the paired and unpaired nucleobases. Substantial stabilisation of RNA bulges can arise from metal ion interactions, where suitable metal binding pockets are created by backbone distortions that have forced negatively charged phosphate groups in the bulge in close proximity [15]. For example, due to the strong interactions between Mg^2+^ ions and adenine-rich bulges found in group I introns, the RNA has been described as folding around a “metal ion core” [16].

This study therefore focused on gaining further insights into how changes in the RNA bulge affect their cleavage by PNAzymes. Subjects of this study included RNA targets **1**–**15**, which contain two recognition motifs for PNAzyme **I** that are identical to those investigated in our previous studies [12] comprising seven canonical base pairs on one side and a wobble base pair closing the bulge, followed by three Watson-Crick pairs on the other side (Table 1). We have previously reported substantial differences between RNAs forming 3- and 4-nucleotide bulges when bound to and cleaved by Zn^2+^-dependent PNAzymes [14], but little is known about 3-nucleotide RNA bulges and Cu^2+^ PNAzymes, which are the most efficient and most selective artificial nucleases reported.

Overall, the rate of the PNAzyme-promoted cleavage of 3-nucleotide RNA bulges in the presence of Cu^2+^ ions was substantially lower than the rates observed with 4-nucleotide RNA bulges. The fastest 4-nucleotide bulge sequences have previously been shown to give half-lives of around 20–30 min [12,13], while the 3-nucleotide bulge-forming RNA sequences were cleaved with approximately 14–24-h half-lives at best. Nonetheless, the cleavage of RNA was highly site-selective and displayed similar tendencies to the previously studied 4-nucleotide bulge systems regarding the nucleotide preferences at specific positions [12]. 

Interestingly, while the general bulge sequence APyPuA (Py referring to pyrimidine and Pu to purine bases) displayed the highest rates of cleavage for 4-nucleotide bulges [12], a similar correlation was observed with the 3-nucleotide bulge systems. Unequivocally, adenosine is the nucleotide best accepted next to the wobble base pair in the 3-nucleotide bulge systems (RNA **1**–**4**, **6**, **10**–**12**; Table 1). The presence of any other nucleotide at that position (RNA **5**, **7**–**9**, **13**–**15**; Table 1) resulted in the extent of RNA cleavage after 24 h being barely above the detection limit. Furthermore, additional similarities can be noted, as either of the purine bases, adenine or guanine (RNA **1**, **3**, **6**; Table 1), were preferred over pyrimidine bases (RNA **2**, **4**, **10**–**12**; Table 1) at the adjacent position. While previously, APyPuA bulges displayed the highest reaction rates, AAPuA resulted in only a slightly lower cleavage efficiency. Likewise, UAA and APuA bulges were the most readily cleaved in the 3-nucleotide bulge system. The overall sequence trends seen with 4-nucleotide bulges appear to be similar for 3-nucleotide bulges. The cleavage of 3-nucleotide RNA bulges occurred in a highly site-selective manner, producing a 2′,3′-cyclic phosphate as the longer fragment, as identified by mass spectrometry. Most importantly, the purine-adenosine cleavage site was preserved and seems to be a minimum requirement for RNA cleavage. Nevertheless, the dramatic decrease in the reaction rate highlights the structural importance of the additional adenosine in 4-nucleotide bulges. 

In general, the preservation of continuous stacking in the double helix is a powerful driving force known to define the structure and interactions of RNA bulges [15]. Moreover, molecular dynamics simulations of an RNA that forms an AAAA bulge closed with a wobble base pair have suggested that the first bulge adenosine on the 5’ side of the bulge is involved in cross-strand stacking interactions, introducing significant rigidity to the structure, whereas the wobble on the other side of the bulge adds flexibility [17]. Therefore, the impact that the bulge adenosine stacking interactions have on the overall conformation and rigidity of the bulge could be substantial. 

### 2.2. Modified Four-Nucleotide Bulge-Forming RNA Targets

In order to gain additional insight into the mechanism and sequence preferences of the efficient cleavage of 4-nucleotide bulge-forming RNA targets, we then performed studies where the cleavage site of the RNA forming an AUAA bulge was modified. The natural, unmodified AUAA bulge (RNA **16**, Table 2) is cleaved to a substantial extent in the presence of PNAzyme **I** and Cu^2+^ ions at pH 7 after only a 1-h reaction time. However, if the adenosine providing the 2′-hydroxyl nucleophile is replaced with an unsubstituted purine riboside lacking the exocyclic amine (RNA **17**, Table 2) to give an AUPurA bulge, the reaction rate drops substantially, increasing the half-life from about 30 min to around 24 h. These findings suggest that the amino group may coordinate to the Cu^2+^ ion and thereby have a role in the positioning of the catalytic ion in critical proximity to the cleavage site. From our previous studies, it is clear that the adenine base can be replaced with guanine without a significant loss in activity, suggesting that the carbonyl functionality on the guanosine can play a similar role to the exocyclic amine on the adenosine. It is also plausible that the pKa of the hydrated metal ion is affected. This would be especially crucial if the mechanism of cleavage involves protonation of the 5′-hydroxyl leaving group by a water molecule bound to the copper ion, as has been suggested for the cleavage of metal aquo ions [18]. 

Moreover, ribose modifications were introduced by replacing the central adenosine with a deoxyadenosine nucleotide (RNA **18**, Table 2) or with a 2′-alkyl modified adenosine (RNA **19**, Table 2). As the 2′-hydroxyl group supplies the nucleophile for the transesterification reaction, these substitutions were expected to result in diminished catalytic activity, which was demonstrated. This is also consistent with the previously reported observation that the immediate product formed upon cleavage by PNAzyme **I** is the 2′,3′-cyclic phosphate [12]. In addition, these results further substantiate the almost complete selectivity of cleavage at a single site, since not even if this site is blocked is the RNA cleaved at an alternate site. Moreover, the possibility to form an RNA/PNAzyme complex with a blocked cleavage site may also allow for the determination of the crystal structure of the complex with Cu^2+^ bound to the neocuproine.

It has been suggested that the activation of phosphodiesters in an RNA strand can be achieved by structural alteration obtained when nucleobases interact with polyaromatic molecules. An example is the introduction of acridine groups in the recognising strand, which makes the RNA target more susceptible to cleavage by lanthanide ions in the vicinity of the acridine moieties and hence gives a higher selectivity. This “pin-point” activation further substantiates that not only the cleaver, but also the structure of the RNA substrate, are crucial elements of the cleavage reaction [19,20]. In order to evaluate if such an effect was partially responsible for the selectivity and high cleavage rate obtained with PNAzyme **I**, we investigated whether the corresponding phenanthrene conjugate would give substantial cleavage of RNA in the presence of external Cu^2+^ ions. Although it cannot be completely excluded, the absence of cleavage with the PNA-phenanthrene conjugate (Table 2, RNA **16** (**Y**), last entry) suggests that pin-point activation does not play a major role in the case of these Cu^2+^ PNAzymes.

### 2.3. PNAzyme II Containing an Extended Recognition Arm

We had asked ourselves if the GCCC hybridising stem, which is the shorter recognition arm of the RNA/PNAzyme complex, retains the duplex conformation during the cleavage of the RNA target. In order to understand the role of this short recognition arm, a new artificial ribonuclease was designed by introducing an extension of two adenine-PNA units to the amino terminus of PNAzyme **I** to give PNAzyme **II**. The principal aim of this investigation was to see whether the PNAzyme activity is retained upon elongation of the RNA/PNAzyme complex. A result to the contrary would suggest that the shorter hybridised region potentially undergoes a structural change critical for the catalytic activity, which would be inhibited by stabilisation of the double strand due to additional base-pairing.

Initially, the ability of the two PNAzymes to promote the cleavage of 4-nucleotide bulge-forming RNAs **16** and **20**–**22** was compared. PNAzyme **I**-promoted cleavage of RNA bulge sequences AUAA and ACAA (RNA **16** and **22**) occurs with half-lives below 30 min in the presence of Cu^2+^ ions at pH 7, while an AAAA bulge (RNA **21**) is cleaved at a slightly lower rate and an AAUA bulge is essentially non-cleavable (RNA **20**, Table 3). Upon complexation of these RNAs with PNAzyme **II**, the extension introduced to the new PNAzyme presents a dangling overhang in relatively close proximity to the cleavage site. The overall trend of cleavage rate dependence on the bulge sequence remained similar to that seen with PNAzyme **I**. Although still displaying efficient cleavage at the same site, the overhang in PNAzyme **II** was shown to reduce the cleavage rates, as illustrated by the decrease in the extent of RNA cleavage after 30 min (Table 3). These observations further demonstrate the specificity of this system, as the delicate molecular environment around the cleavage site can be disturbed by overhanging nucleotides in the case of off-target RNA sequences with incomplete recognition arms. This may become important as it could further increase the specificity of PNAzymes.

**Figure 1:**
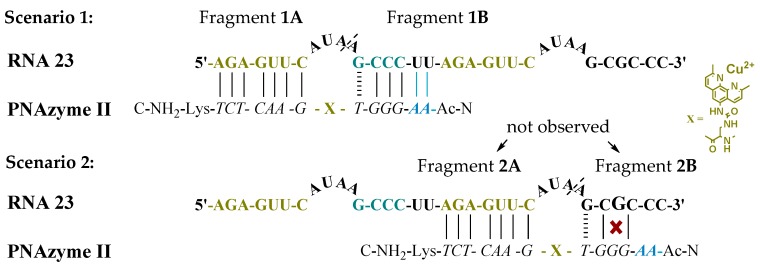
Possible complexes between RNA **23** and PNAzyme **II.** The dashed line denotes the cleavage site.

Moreover, the cleavage of an extended RNA target was studied (RNA **23**, Figure 1), where the extension present in PNAzyme **II** is able to form additional Watson-Crick base pairs, with the UU sequence in the RNA preceding the short recognition arm. As such, compared to the complex with RNA **16**, four additional hydrogen bonds will be formed with RNA **23**, stabilising the duplex stem (Figure 1, Scenario 1). In addition to containing the RNA **16** sequence with an extended short recognition arm, RNA **23** also contains a competing long recognition arm, the potential to form the fastest cleaved bulge and a mismatch-containing sequence that otherwise resembles the short recognition arm (Figure 1, Scenario 2). 

As previously shown, a single mismatch in the short arm of RNA **16** leads to a very low cleavage rate with PNAzyme **I**, [12] and the cleavage of RNA **23** occurs exclusively at the bulge formed between the matching recognition arms, producing fragments **1A** and **1B** (Figure 1). This demonstrates excellent mismatch discrimination. While PNAzyme **I**-promoted cleavage of this long RNA **23** target is qualitatively analogous to the cleavage of RNA **16**, the cleavage of RNA **23** seemed to occur at a somewhat lower rate (Table 4). In contrast, the extended PNAzyme **II**-promoted cleavage of RNA **23** occurred at a higher rate than with PNAzyme **I**. In fact, the improved rate was essentially identical to that of PNAzyme **I**-promoted cleavage of RNA **16**. Therefore, not only did the extended recognition arm leave the catalytic activity undisturbed, but it allowed for the fastest cleavage rate to be retained for the first time on a longer, more complex RNA target which resembles a more likely natural target. 

**Figure 2:**
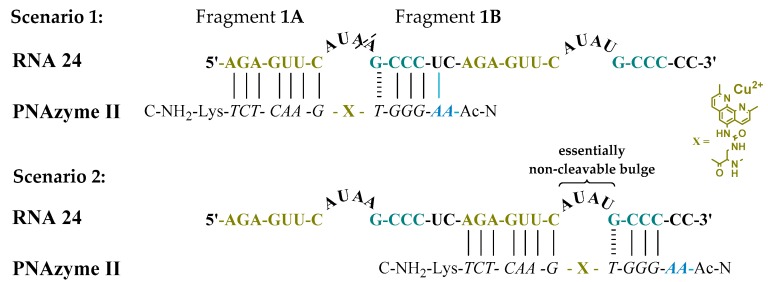
Possible complexes between RNA **24** and PNAzyme **II.** The dashed line denotes the cleavage site.

Moreover, the cleavage of another long RNA sequence (RNA **24**, Figure 2) was studied, where the same short recognition arm was only extended by one nucleobase instead of two. In addition, RNA **24** contained an alternative set of recognition arms further along the sequence, surrounding a bulge sequence that renders the system catalytically inactive [12]. Crucially, now, the alternative binding scenario provides more competition than it did in the case of RNA **23**, due to the absence of the mismatch, while a competing cleavage site is missing and thus only site-specific cleavage producing fragments **1A** and **1B** can occur. As expected, the extent of PNAzyme **II** promoted RNA cleavage after 30 min decreased (RNA **24**, Table 4), possibly due to competitive binding to the non-productive site and/or due to lower activity with PNA overhang.

**Figure 3:**
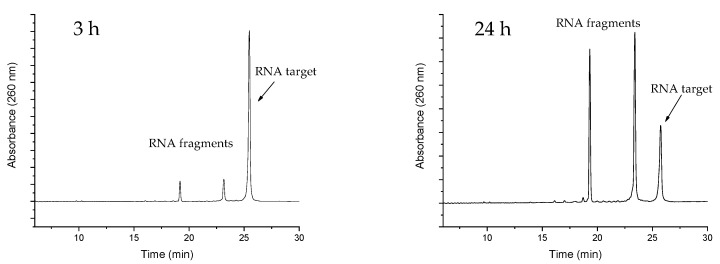
Representative IE HPLC chromatograms showing the extent of RNA cleavage after a 3- and 24-h reaction time in the presence of sub-stoichiometric PNAzyme **II**. The experiments were performed with a 10-fold excess of RNA **23** (4.0 µM) with respect to PNAzyme **II** (0.4 µM) in the presence of Cu^2+^ (10 µM) at 37 °C, pH 7.0, in HEPES buffer (10 mM HEPES, 0.1 M NaCl).

Finally, the ability to give turnover of the substrate (and thus exhibit true catalytic behaviour) is a crucial feature of an artificial enzyme. As such, the overall potency of an artificial ribonuclease depends upon its overall affinity to the target, but also on its ability to release the cleaved RNA fragments, which then allows for it to bind to and trigger the cleavage of the next target. Thus, the potency of a catalytic antisense oligonucleotide could in fact be reduced by overly strong binding to the target. As efficient turnover of RNA **16** by PNAzyme **I** has been previously demonstrated [12], it then became critical to investigate whether the additional base-pairs in the RNA **23**/PNAzyme **II** complex may enhance the binding to such a degree as to impair turnover. This was shown not to be the case in experiments with sub-stoichiometric PNAzyme **II**. Multiple turnover was clearly demonstrated after a 24-h period with a 10-fold excess of RNA **23** (Figure 3).

## 3. Materials and Methods

Peptide nucleic acid monomers, Fmoc-PNA-A(Bhoc)-OH, Fmoc-PNA-G(Bhoc)-OH, Fmoc-PNA-C(Bhoc)-OH and Fmoc-PNA-T-OH, were purchased from Link Technologies Ltd. (Glasgow, UK). Rink Amide resin (ChemMatrix, 0.47 mmol/g) was purchased from Biotage (Uppsala, Sweden). Fmoc-l-Dap(Mtt)-OH (2,3-diaminopropionic acid) and Fmoc-l-Lys(Boc)-OH were purchased from Iris Biotech GmbH (Marktredwitz, Germany). 9-aminophenanthrene was purchased from Sigma Aldrich (St. Louis, MO, USA). Ethyl cyano(hydroxyimino) acetate (Oxyma) and *N*,*N*′-diisopropylcarbodiimide (DIC) were purchased from Merck-Millipore (Burlington, MA, USA). All other reagents and solvents used were of analytical commercial quality. All chemicals used in the RNA cleavage assays were of molecular biology grade. All RNA oligomers were purchased from Dharmacon (Lafayette, CO, USA). RNA oligomers 5′-AGAGUUCAAAGCCC-3′ (RNA **1**) and 5′-AGAGUUCAUAGCCC-3′ (RNA **2**) were purchased purified and all other RNAs were deprotected according to the manufacturer´s protocol (2′-ACE protecting groups), purified by Ion-Exchange High Performance Liquid Chromatography (IE-HPLC) and desalted by Reversed Phase HPLC (RP-HPLC). IE-HPLC purifications were performed using an analytical Thermo Scientific DNAPac PA-100 BioLC (4 × 250 mm) column (Waltham, MA, USA) with UV detection at 260 nm. A linear gradient of 0–35% buffer B over 15 min was used with a flow rate of 1.5 mL/min at 60 °C, (buffer A) 20 mM NaOAc in 30% MeCN/aq., and (buffer B) 20 mM NaOAc in 0.4 M LiClO_4_ in 30% MeCN/aq. Purified RNAs were desalted by reverse-phase HPLC on a Jasco HPLC system using the Supelco Discovery BIO Wide Pore C18-5.5 μm (250 × 4.6 mm) column (Sigma-Aldrich, St. Louis, MO, USA). A linear gradient of 0–37 % buffer B over 20 min was used with a flow rate of 1 mL/min at 50 °C, (buffer A) 50 mM triethylammonium acetate (TEAA) in water (pH 6.5) and (buffer B) 50 mM TEAA in water (pH 6.5)-acetonitrile (1:1, *v*/*v*). Purified and desalted RNAs were lyophilised three times before use and stored as frozen solutions. Concentrations of RNA sequences were determined by UV absorption at 260 nm on a Varian Cary 300 UV-Vis dual beam spectrophotometer (Varian, Palo Alto, CA, USA) and calculated from extinction coefficients obtained by the nearest neighbour approximation [21].

### 3.1. Synthesis of Peptide Nucleic Acid-Based Artificial Ribonucleases (**PNAzymes I and II**) and the Peptide Nucleic Acid-Phenanthrene Conjugate

PNAzyme **I** was prepared according to previously reported method 1 [22] or method 2 [14]. The PNA-phenanthrene conjugate was prepared following method 2 [14], except for the post-conjugation step, where the 9-aminophenanthrene precursor was used instead of 5-amino-2,9-dimethyl-1,10-phenanthroline. The chimeric PNA sequence of PNAzyme **II** was assembled automatically as previously reported [14]. Post-conjugation of phenyl *N*-(2,9-dimethyl-1,10-phenantrolin-5-yl)carbamate, synthesised as previously reported [8], to the resin-bound PNA, was performed according to a reported procedure [22]. The conjugate was then cleaved from the support by treatment with TFA/water/TIS (95:2.5:2.5, *v*/*v*/*v*) over a 2-h period. The conjugated PNA product was evaporated to dryness by a nitrogen flow, diluted with deionised (MilliQ) water and evaporated to dryness under reduced pressure. The crude product was partitioned between diethyl ether and water (×3), and the water phase was evaporated to dryness. 

RP-HPLC purification and analysis was carried out on an Ascentis Express Supelco Peptide ES-C18 column (2.7 μm, 150 × 4.6 mm) with a linear gradient elution of 0% to 40% buffer B over 30 min at 60 °C, using a flow rate of 1.0 mL/min and UV detection at 260 nm (corresponding chromatograms shown in Figure S5). Improved separation was achieved with a gradient of 0 to 10% buffer B in 2 min, followed by 10 to 20% buffer B from 2 to 10 min, and 20 to 25% from 10 to 20 min. The following solvent system was used: solvent A: 0.1% TFA in water; solvent B: 50% MeCN: water containing 0.1% TFA. Collected products were evaporated to dryness and lyophilised from water (×3). The final products were analysed by MALDI-MS in positive ion-mode using a sinapic acid matrix (10 mg/mL in 0.1% TFA/milliQ and MeCN (2:1, *v/v*), *m/z*: PNAzyme **I** C_145_H_180_N_71_O_38_ [M]^+1^ calcd 3538.4, found 3538.4; *m/z*: PNA-phenanthrene conjugate C_145_H_178_N_70_O_38_ [M]^+1^ calcd 3508.4, found 3508.8; *m/z*: PNAzyme **II** C_167_H_206_N_86_O_42_ [M]^+1^ calcd 4088.7, found 4087.0. Concentrations of PNA conjugates were determined by UV absorption at 260 nm on a Varian Cary 300 UV-Vis dual beam spectrophotometer (Varian) and calculated from extinction coefficients obtained by the nearest neighbour approximation [21]. Representative RP HPLC chromatograms for the reference unconjugated PNA **II**, crude PNAzyme **II** and purified PNAzyme **II** are available in the supplementary material.

### 3.2. RNA Cleavage Assay Using Anion Exchange (IE) HPLC

RNA cleavage reactions were carried out in sealed tubes immersed in a thermostated water bath (37 °C). Experiments were performed at pH 7.4 (RNAs **1**–**15**) or pH 7.0 (RNAs **16**–**24**) in HEPES buffer (10 mM HEPES, 0.1 M NaCl). RNA targets (4 µM final concentration) were equilibrated in appropriate amounts of water and HEPES buffer over a 15-minute period at 37 °C prior to the addition of Cu^2+^ solution (10 µM final concentration) and the PNAzyme (or PNA-phenanthrene conjugate, 1.0 equiv, except for PNAzyme **I** and **II** comparison studies, where 1.3 equiv of either PNAzyme was used, and except for the PNAzyme **II** turnover studies where 0.1 equiv was used). The reaction mixtures were then allowed to incubate at 37 °C. Aliquots (20 or 40 µL) were withdrawn at specified time points and immediately quenched (70 or 90 µL of 300–600 µM EDTA in 30% MeCN/milliQ). The samples were analysed by anion exchange HPLC (IE-HPLC) using a Dionex NucleoPac PA-100 (4 × 250 mm) column with a linear gradient elution of 0–45% buffer B over 30 min at 60 °C. A flow rate of 1.5 mL/min was used and UV detection was carried out at 260 nm. The following solvent system was used: (A) 20 mM NaOAc in 30% aq. MeCN; and (B) 20 mM NaOAc, 0.4 M LiClO_4_ in 30% aq. MeCN. Cleavage of RNA substrates was obtained by the quantification of the remaining RNA and the sum of the formed fragments detected in the IE-HPLC analysis. Representative chromatograms can be found in the supplementary material.

### 3.3. Determination of RNA Cleavage Sites

RNA cleavage reactions were performed as above. The main RNA fragments were collected from IE-HPLC runs and desalted as described earlier. The collected fractions were analysed by ES mass spectrometry in negative ion mode using a solution of MeCN–water (1:1, *v*/*v*)**,** *m*/*z* RNA **3** (5′-AGA-GUUC-AGA-GCCC-3′) longer fragment (5′-AGA-GUUC-AG-3′ cyclic phosphate) C_87_H_104_N_37_O_62_P_9_ [M]^−2^ calcd 1468.7, found 1469.1; *m*/*z* RNA **6** (5′-AGA-GUUC-UAA-GCCC-3′) longer fragment (5′-AGA-GUUC-UA-3′ cyclic phosphate) C_86_H_103_N_34_O_63_P_9_ [M]^−2^ calcd 1449.2, found 1449.6. These MS spectra are available in the supplementary material.

## 4. Conclusions

Cu^2+^-dependent PNAzyme-promoted cleavage of RNA target sequences was studied with respect to the bulge length, the bulge composition and the length of the shorter hybridising recognition arm. PNAzyme-promoted cleavage of RNA target sequences, where 3-nucleotide bulges are formed, was investigated and the cleavage rates compared for different bulge compositions. PNAzyme-promoted RNA cleavage of 3-nucleotide RNA bulges occurred at significantly lower rates than the corresponding 4-nucleotide bulge systems. The nucleotide preferences in the bulge were, nonetheless, similar in both systems, as AUAA and UAA bulges were cleaved most efficiently in the respective systems. Essentially no cleavage occurred when the 2′-hydroxyl nucleophile of the central adenosine in the 4-nucleotide AUAA bulge was absent, as in the case of 2′-*O*-methyladenosine or deoxyadenosine, which further demonstrates the site-specific nature of RNA cleavage by the investigated Cu^2+^ PNAzymes. Furthermore, the exocyclic amino group of the central nucleophilic adenosine in the AUAA bulge was shown to be critical for the catalytic activity of the PNAzyme, as the reaction rate dropped drastically upon its exclusion from the structure. Thus, some mechanistic insights were derived, as the exocyclic amine could have a role in coordinating the copper ion, which could also occur with the carbonyl group in the case of a guanosine nucleotide at that position. Although we still do not have the full picture which allows for the elucidation of a detailed mechanism of cleavage with the studied Cu^2+^ PNAzymes, there are data and previous interpretations that allow some speculation. It is feasible that the copper ion coordinates the cleaved phosphodiester, while at the same time, a copper bound water molecule protonates the leaving 5’-oxygen, forming a favourable six membered ring (P-O-Cu-O-H-O-5), as suggested for metal aquo ions in solutions [18]. It may be geometrically feasible that the copper ion could coordinate the N^6^ of A or O^6^ of G (possibly via a Cu-bound water molecule) at the same time as coordinating the phosphate and via water, protonating the leaving 5’-oxygen, but it is likely that a substantial conformational change would be needed. 

Finally, the shorter RNA/PNAzyme recognition arm was extended by two nucleobases in order to examine whether the ease of dissociation might correlate with the cleavage rate. The extended PNAzyme was shown to perform at an even higher rate when fully hybridised to a longer RNA target, compared to the previous shorter PNAzyme. All in all, the study has added valuable mechanistic insights into the action of Cu^2+^-dependent PNA-based artificial ribonucleases and serves as a basis for further developments. For an RNA target that is longer than the PNAzyme, and thus more closely resembling a natural substrate, the highest cleavage rate so far was reported, and the artificial enzyme was shown to give turnover of the RNA substrate.

## Figures and Tables

**Table 1:** The extent of cleavage of 3-nucleotide bulge-forming RNA sequences **1**–**15** after incubation in the presence of equimolar PNAzyme **I** over a period of 3 h and 24 h. ^1^

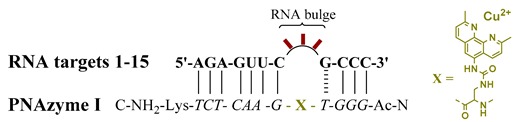				
RNA	RNA Bulge Sequence	% RNA Cleaved		
		3 h	24 h	
**1**	-**AAA**-	18	56	
**2**	-**AUA**-	6	19	
**3**	-**AGA**-	14	49	
**4**	-**ACA**-	3	11	
**5**	-**AAU**-	<2	3	
**6**	-**UAA**-	19	69	
**7**	-**AUG**-	<2	6	
**8**	-**AUC**-	<2	<2	
**9**	-**AUU**-	<2	2	
**10**	-**GUA**-	3	13	
**11**	-**CUA**-	4	19	
**12**	-**UUA**-	6	28	
**13**	-**GUG**-	<2	5	
**14**	-**CUC**-	<2	3	
**15**	-**ACG**-	<2	4	

^1^ Experiments were performed with RNAs **1**–**15** (4 µM) incubated in the presence of PNAzyme I (1.0 equiv) and Cu^2+^ (10 µM) at 37 °C, pH 7.4, in HEPES buffer (10 mM HEPES, 0.1 M NaCl). The % RNA cleavage values are average values of at least two experiments with a standard error of less than ± 2.

**Table 2:** The extent of cleavage of 4-nucleotide bulge-forming reference RNA **16** and modified RNA sequences **17**–**19** after incubation in the presence of equimolar PNAzyme **I** over a period of 1 h, 3 h and 24 h. ^2^

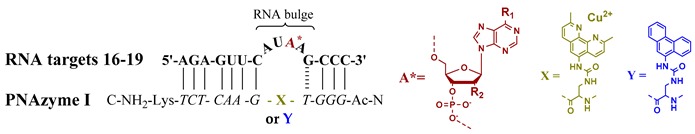							
RNA	Conjugate	Bulge Modification (A*)	R_1_	R_2_	% RNA Cleaved		
					1 h	3 h	24 h
**16**	**X**	Unmodified adenosine	NH_2_	OH	71		
**17**	**X**	Purine	H	OH		15	46
**18**	**X**	Deoxyadenosine	NH_2_	H		1	5
**19**	**X**	2′-*O*-Methyladenosine	NH_2_	OMe		3	7
**16**	**Y**	Unmodified adenosine	NH_2_	OH		n.d.	n.d.

^2^ Experiments were performed with RNAs **16**–**19** (4 µM) incubated in the presence of PNAzyme **I** or the corresponding phenanthrene conjugate (Y) (1.0 equiv) and Cu^2+^ (10 µM) at 37 °C, pH 7.0, in HEPES buffer (10 mM HEPES, 0.1 M NaCl). n.d. = not detected. The % RNA cleavage values are average values of at least two experiments with a standard error of less than ± 2.

**Table 3:** Schematic representation of complexes between 4-nucleotide bulge-forming RNA sequences **20**–**22** and PNAzyme I or II, followed by the extent of cleavage of RNAs **16** and **20**–**22** after incubation in the presence of PNAzyme **I** or **II** over a period of 30 min. ^3^

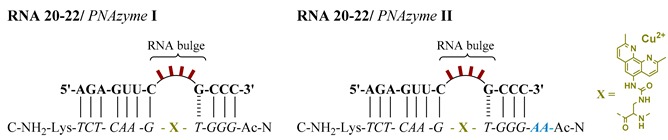			
RNA	Bulge Sequence	% RNA Cleaved after 30 min	
		PNAzyme I	PNAzyme II
**16**	-**AUAA**-	58	40
**20**	-**AAUA**-	2	<2
**21**	-**AAAA**-	36	23
**22**	-**ACAA**-	54	45

^3^ Experiments were performed with RNAs **16** and **20**–**22** (4 µM) incubated in the presence of PNAzyme I or II (1.3 equiv) and Cu^2+^ (10 µM) at 37 °C, pH 7.0, in HEPES buffer (10 mM HEPES, 0.1 M NaCl). The % RNA cleavage values are average values of at least two experiments with a standard error of less than ± 2.

**Table 4:** The extent of cleavage of RNAs **23** and **24** after incubation in the presence of PNAzyme **I** or **II** over a period of 30 min and 1 h. ^4^

RNA	PNAzyme	% RNA Cleaved	
		30 min	1 h
**23**	**I**	37	55
	**II**	59	79
**24**	**II**	37	54

^4^ Experiments were performed with RNA (4 µM) incubated in the presence of PNAzyme **I** or **II** (1.3 equiv) and Cu^2+^ (10 µM) at 37 °C, pH 7.0, in HEPES buffer (10 mM HEPES, 0.1 M NaCl). The % RNA cleavage values are average values of two experiments with a standard error of no more than ± 2 for RNA 23 and ± 5 for RNA 24.

## Supplementary Materials

The following are available online, Figure S1: Ion exchange HPLC chromatograms of PNAzyme **I**-assisted cleavage of 3-nucleotide bulge-forming RNAs **1**–**15** (24 h). Figure S2: MS spectra of fragments (2′,3′-cyclic phosphates) produced in the cleavage of RNA **3** and **6**. Figure S3: Ion exchange HPLC chromatograms of PNAzyme-**I** assisted cleavage of RNA **16** (1 h) and 4-nucleotide bulge-forming modified RNAs **17**–**19** (24 h) and the absence of cleavage of RNA **16** in the presence of PNA-phenantroline conjugate (**Y**) (24 h). Figure S4: IE HPLC chromatograms of cleavage of 4-nucleotide bulge-forming RNAs **16, 20**–**24** as promoted by PNAzyme **I** or **II** (30 min). Figure S5: Reversed-phase HPLC chromatograms representative of analysis and purification of PNAzyme **II**.
