# Supplementary material for: Further Probing of Cu2+-Dependent PNAzymes Acting as Artificial RNA Restriction Enzymes
Source: Molecules. 2019 Feb 14;24(4):672. doi: 10.3390/molecules24040672 (PMC6412939; doi:10.3390/molecules24040672)

Supplementary Information to

Further Probing of Cu^{2+}-Dependent PNAzymes Acting as Artificial RNA Restriction Enzymes

Olivia Luige, Merita Murtola, Alice Ghidini and Roger Strömberg

Representative chromatograms of the following HPLC analyses are included (time points at which the aliquots were collected indicated in parentheses):

S1 Cleavage of 3-nucleotide bulge-forming RNAs **1-15** by PNAzyme **I** (24 h)

S2 ESI MS spectra of fragments (2',3'-cyclic phosphates) produced in the cleavage of RNA **3** and **6**

S3 Cleavage of (or the lack thereof) modified 4-nucleotide bulge-forming RNAs **16 (**1 h) and **17**-**19** by PNAzyme **I** (24 h) and RNA **16** in the presence of PNA-phenanthrene conjugate (**Y**) (24 h)

S4 Cleavage of 4-nucleotide bulge-forming RNAs **16**, **20**-**24** (30 min) promoted by PNAzyme **I** or **II**

S5 RP HPLC analysis of PNAzyme **II**

S1. Cleavage of 3-nucleotide RNA bulge-forming RNAs **1-15** by PNAzyme **I** (24 h)

RNA **1** (5′-AAA-3′)

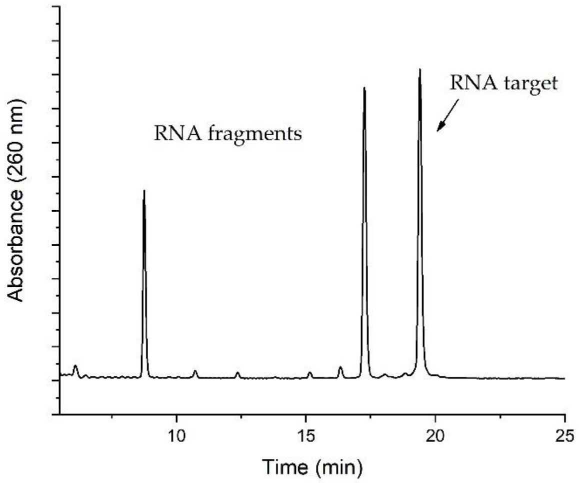

RNA **2** (5′-AUA-3′)

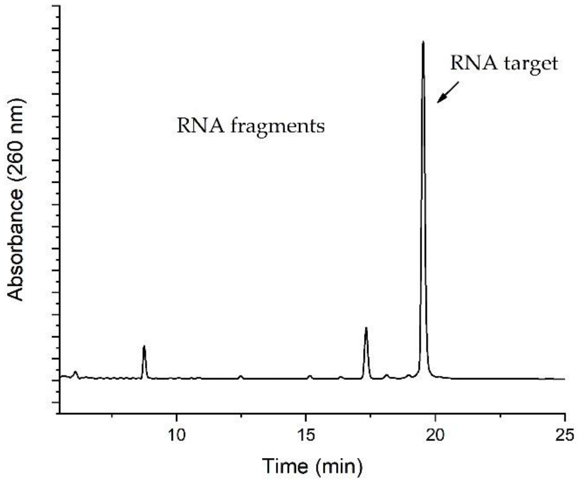

RNA **3** (5′-AGA-3′)

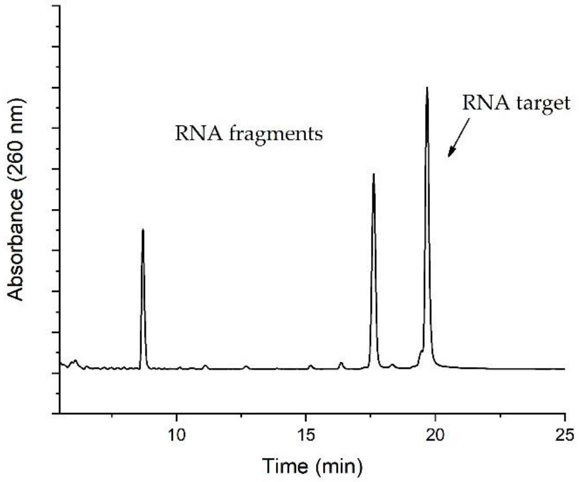

RNA **4** (5′-ACA-3′)

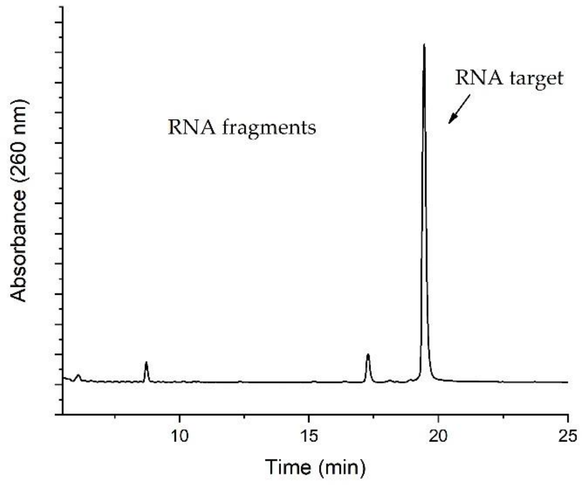

RNA **5** (5′-AAU-3′)

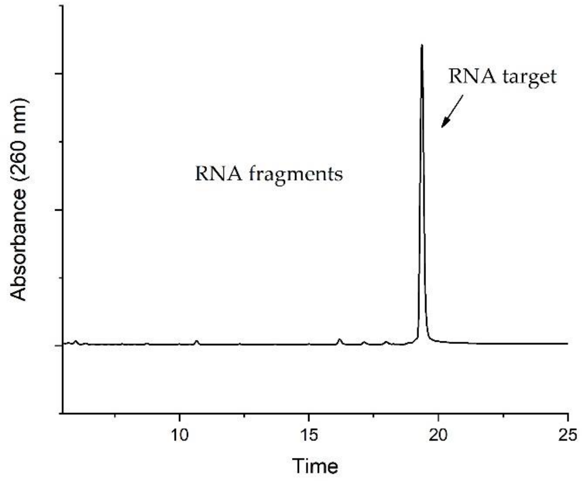

RNA **6** (5′-UAA-3′)

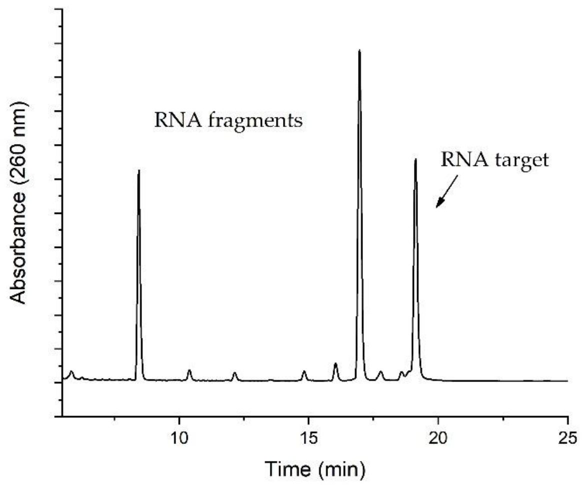

RNA **7** (5′-AUG-3′)

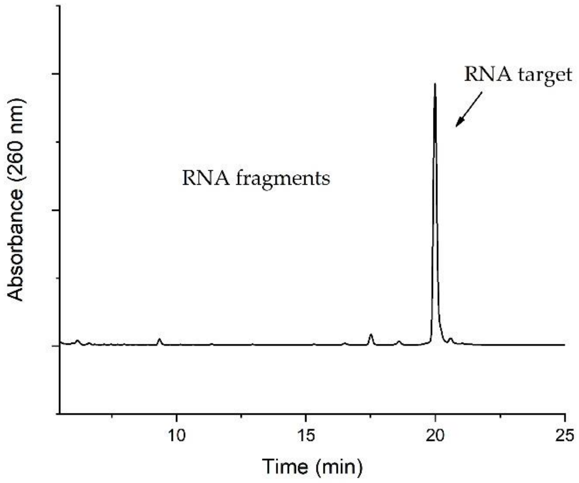

RNA **8** (5′-AUC-3′)

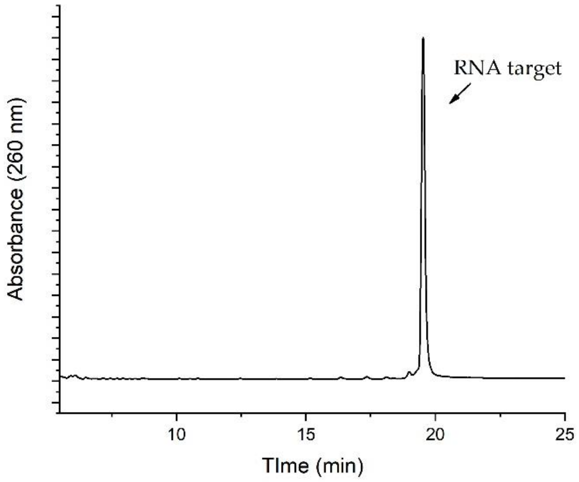

RNA **9** (5′-AUU-3′)

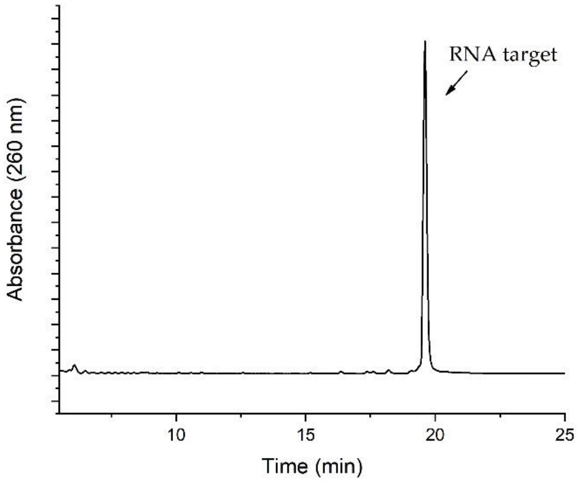

RNA **10** (5′-GUA-3′)

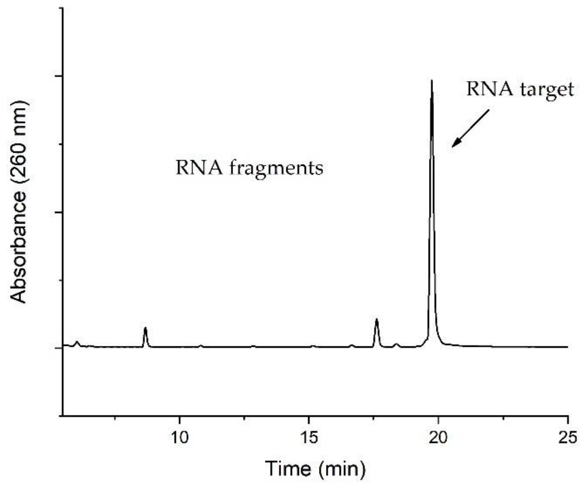

RNA **11** (5′-CUA-3′)

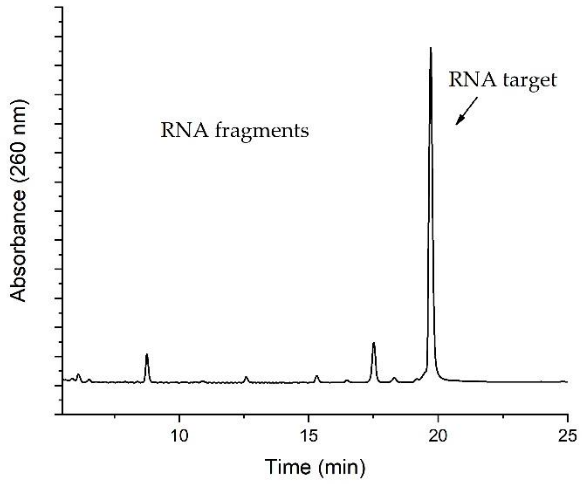

RNA **12** (5′-UUA-3′)

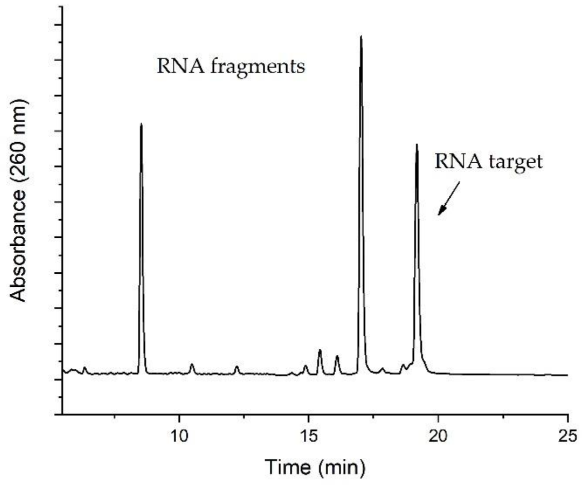

RNA **13** (5′-GUG-3′)

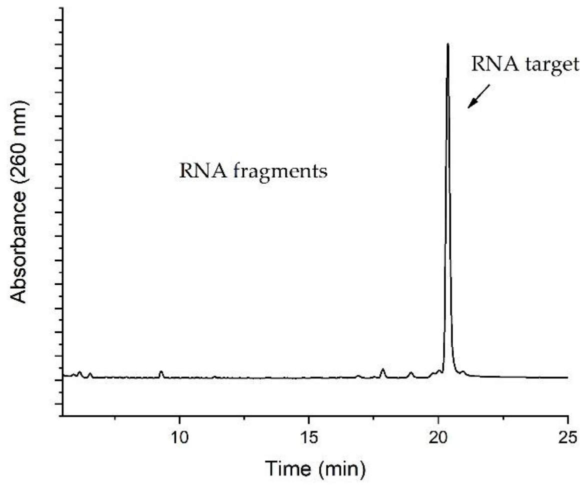

RNA **14** (5′-CUC-3′)

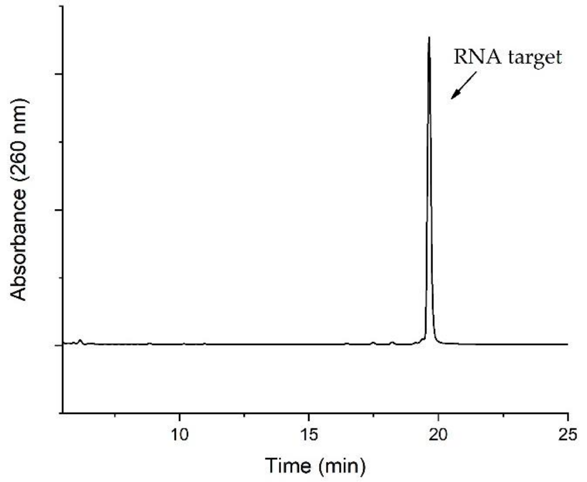

RNA **15** (5′-ACG-3′)

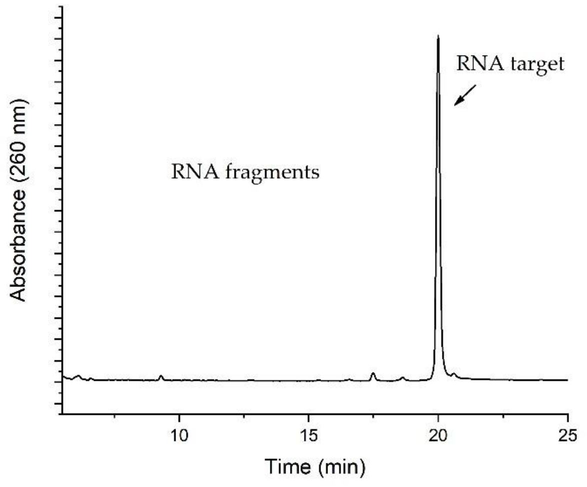

S2 ES MS spectra of fragments (2',3'-cyclic phosphates) produced in the cleavage of RNA **3** and **6**

RNA **3** fragment 5′-AGAGUUC-AG-3′ cyclic phosphate

m/z: $C_{87}H_{104}N_{37}O_{62}P_9^{2-}$ $[M]^{-2}$ calcd 1468.7, found 1469.1

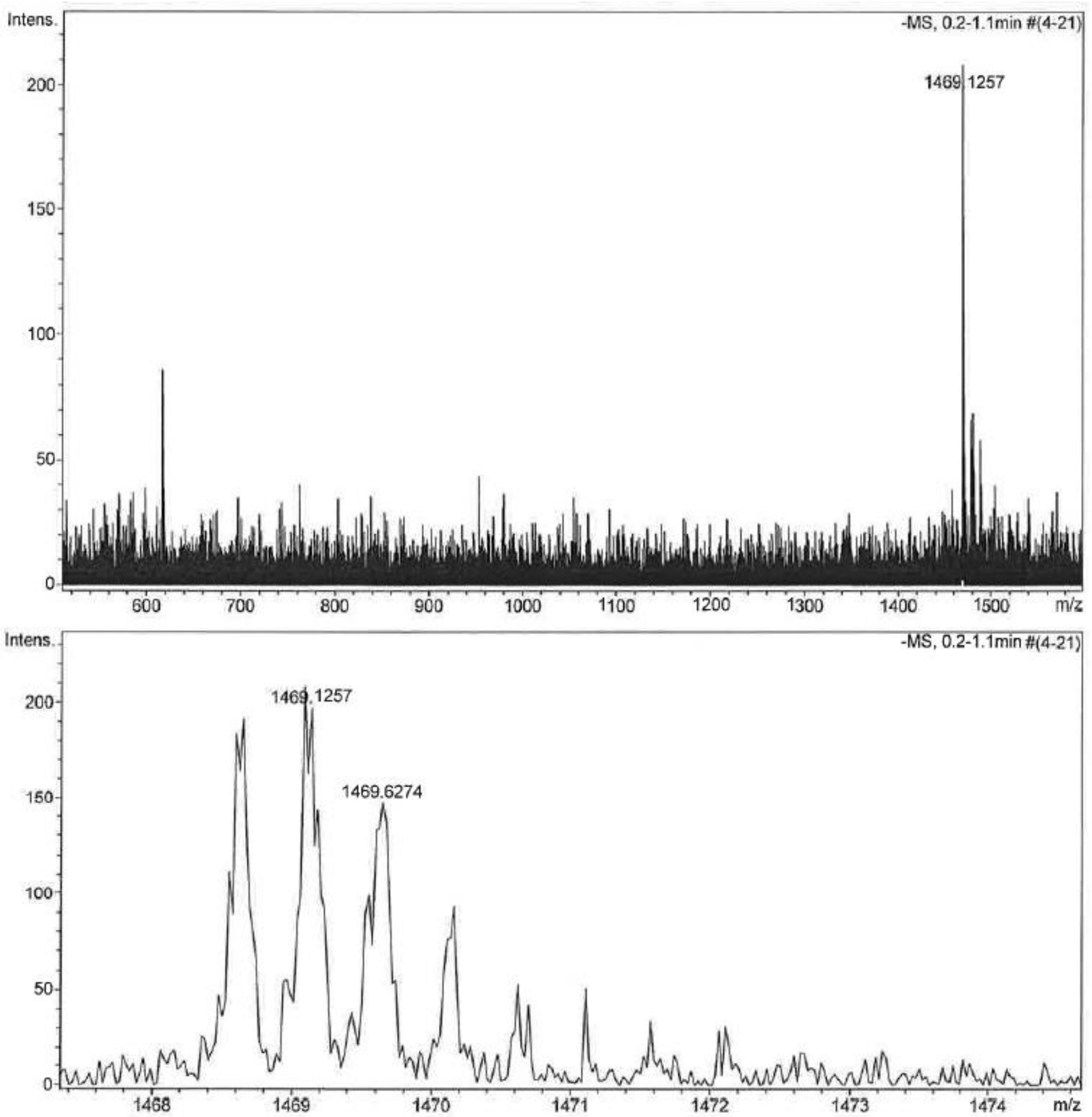

RNA **6** fragment 5′-AGAGUUC-UA-3′ cyclic phosphate

m/z: $C_{86}H_{103}N_{34}O_{63}P_9^{2-}$ $[M]^{-2}$ calcd 1449.2, found 1449.6

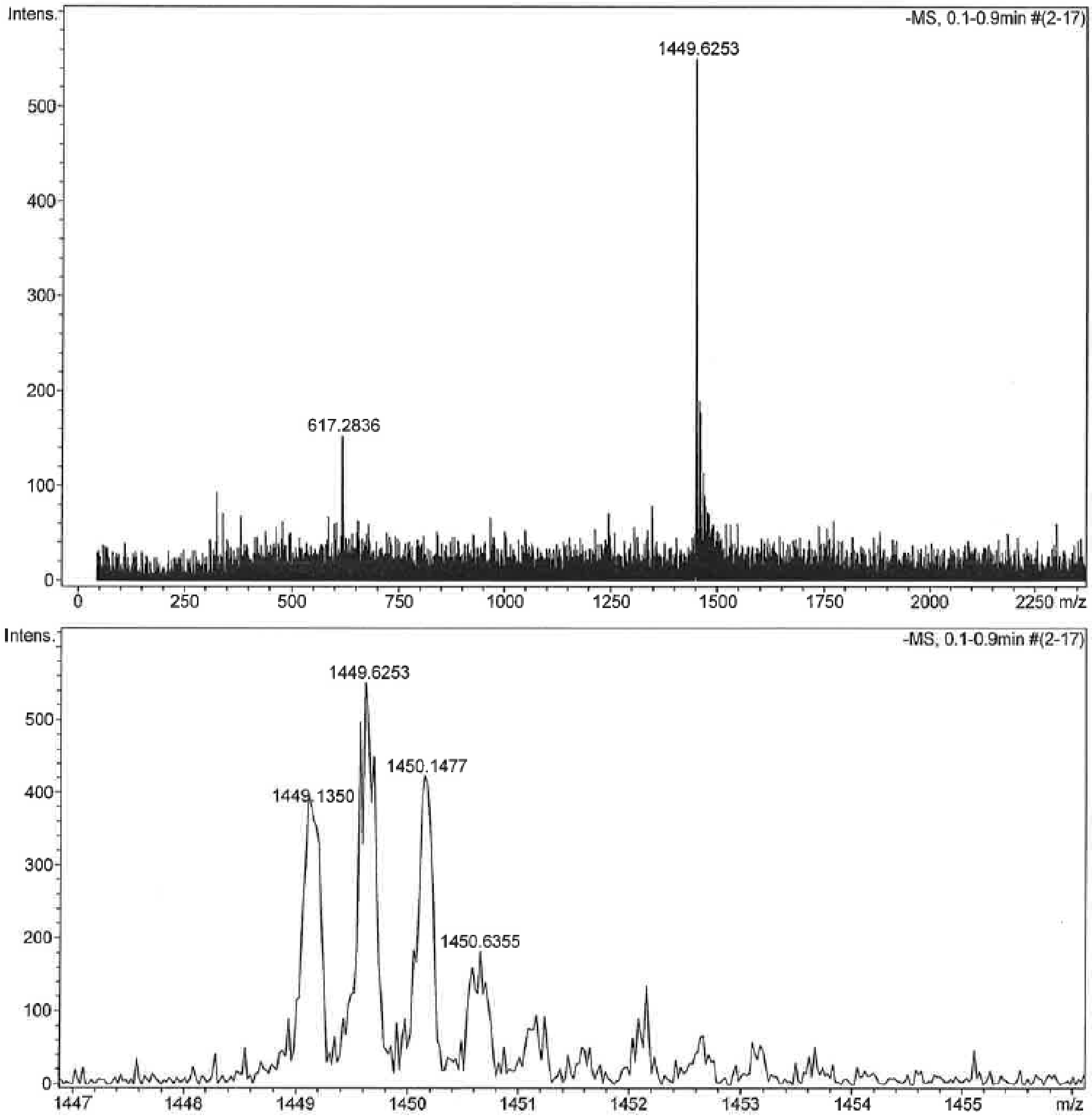

S3 Cleavage of (or the lack thereof) modified 4-nucleotide bulge-forming RNAs **16** (1 h) and **17-19** (24 h) by PNAzyme **I**, and RNA **16** in the presence of PNA-phenanthrene conjugate (**Y**) (24 h)

RNA **16** (5′-AUAA-3′)

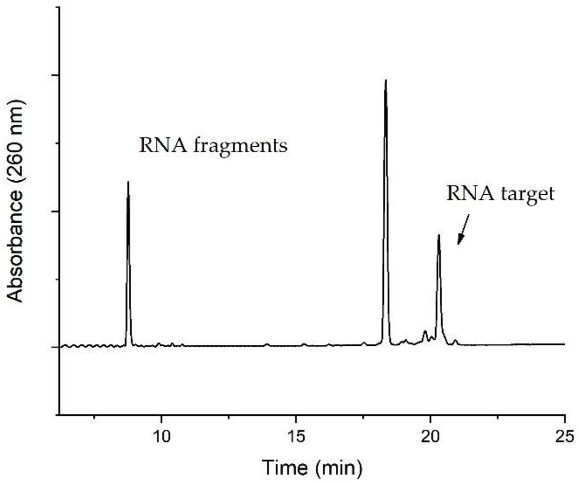

RNA **17** (5′-AUPurA-3′)

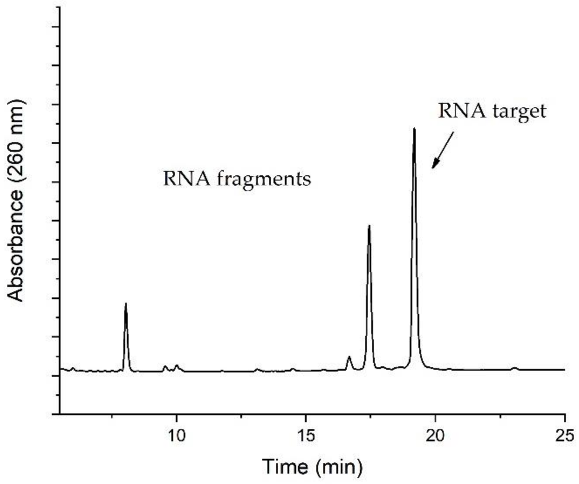

RNA **18** (5′-AUdAA-3′)

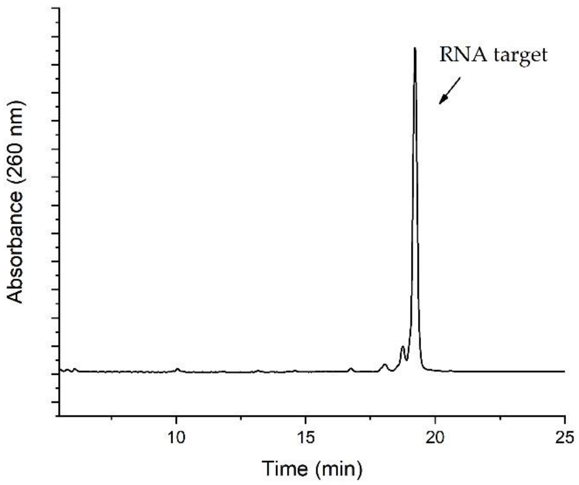

RNA **19** (5′-AUA$_{2'OMe}$A-3′)

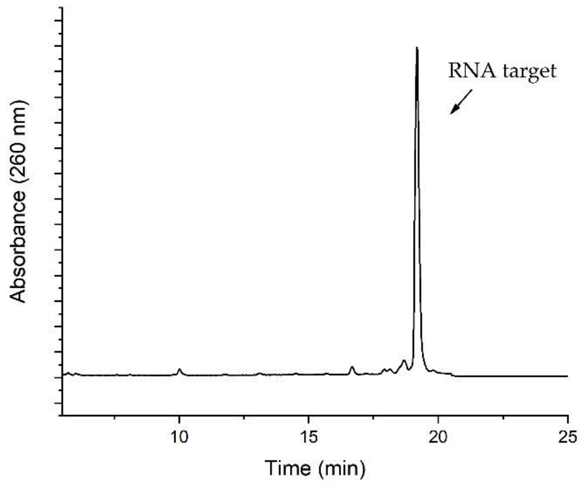

RNA **16** and PNA-phenanthrene conjugate (**Y**)

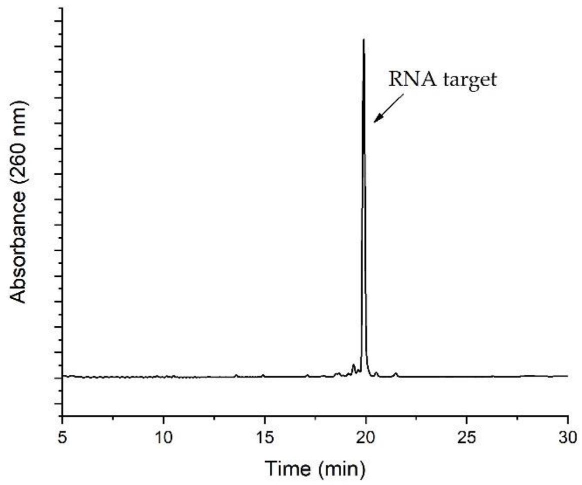

S4 Cleavage of 4-nucleotides RNAs **16, 20-24** promoted by PNAzyme **I** or **II** (30 min)

PNAzyme **I**-promoted cleavage of RNA **16** (5′-AUAA-3′)

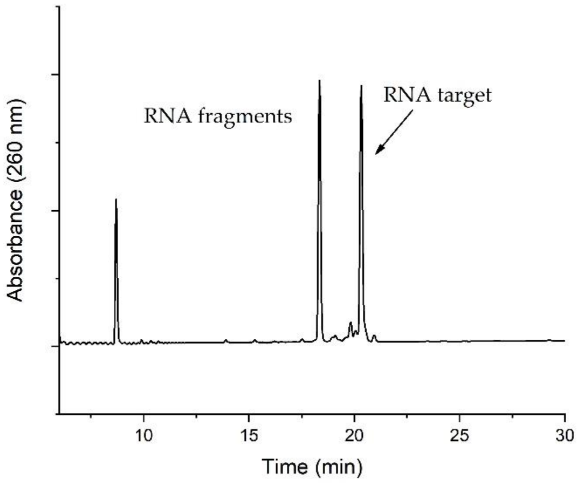

PNAzyme **II**-promoted cleavage of RNA **16** (5′-AUAA-3′)

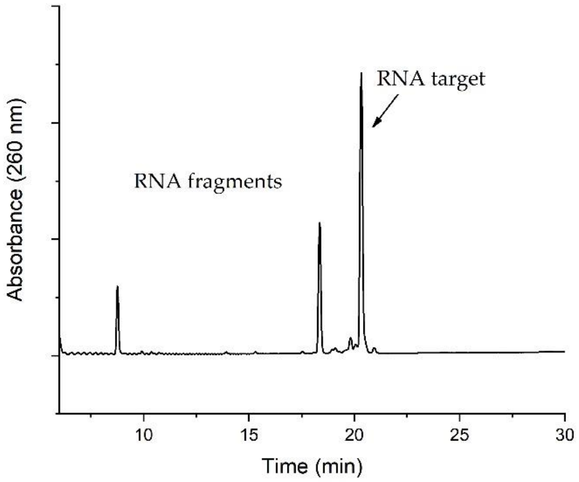

PNAzyme **I**-promoted cleavage of RNA **20** (5′-AAUA-3′)

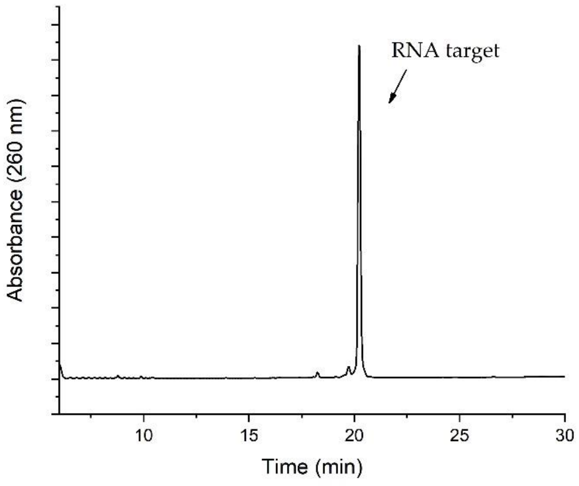

PNAzyme **II**-promoted cleavage of RNA **20** (5′-AAUA-3′)

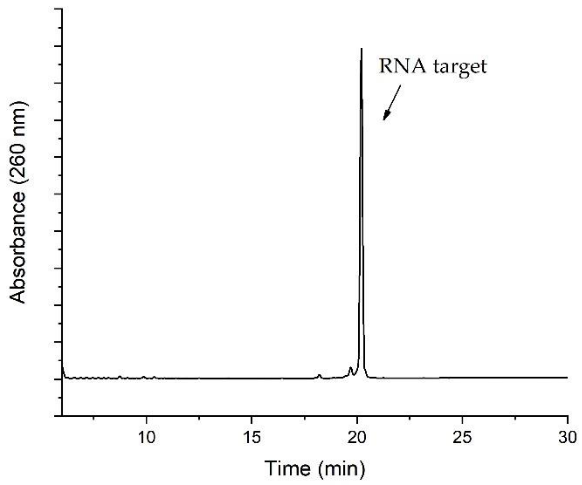

PNAzyme **I**-promoted cleavage of RNA **21** (5′-AAAA-3′)

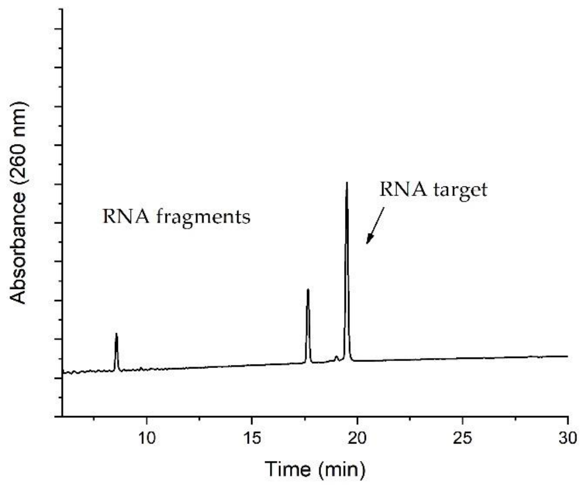

PNAzyme **II**-promoted cleavage of RNA **21** (5′-AAAA-3′)

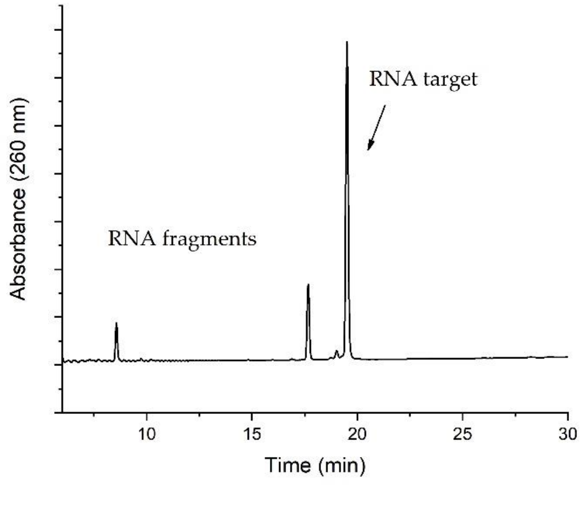

PNAzyme **I**-promoted cleavage of RNA **22** (5′-ACAA-3′)

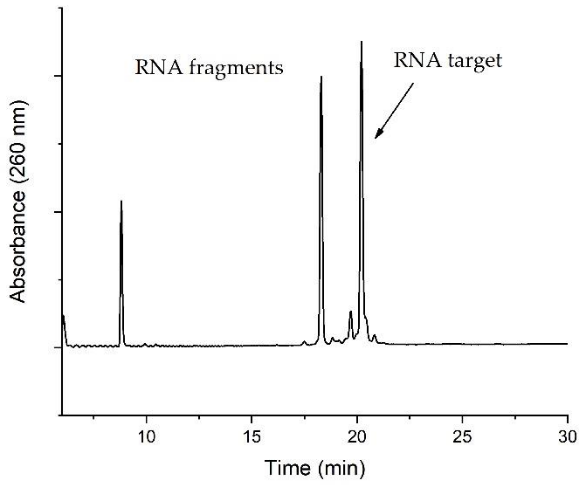

PNAzyme **II**-promoted cleavage of RNA **22** (5′-ACAA-3′)

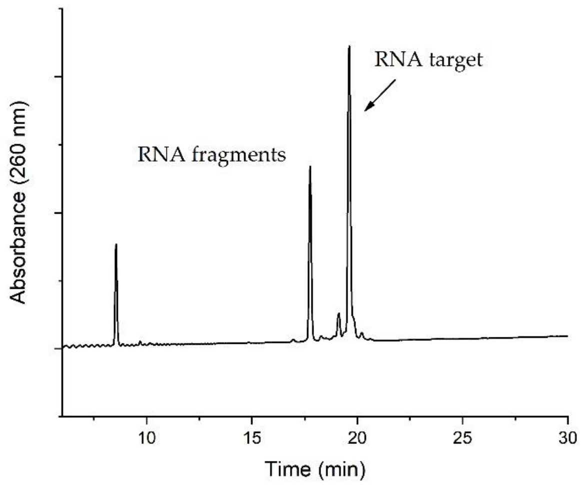

PNAzyme **I**-promoted cleavage of RNA **23** (36mer 5′-AUAA-3′)

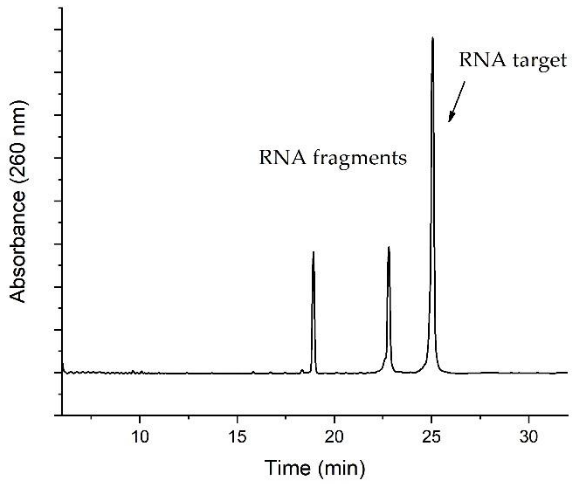

PNAzyme **II**-promoted cleavage of RNA **23** (36mer 5′-AUAA-3′)

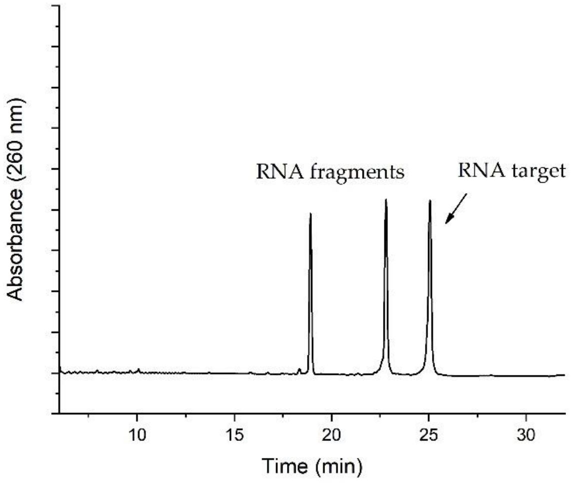

PNAzyme **II**-promoted cleavage of RNA **24** (36mer 5′-AUAA-3′ and 5′-AUAU-3′)

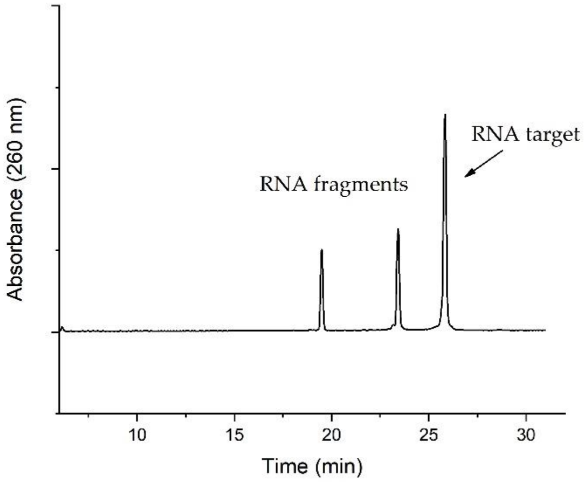

S5 RP HPLC analysis of PNAzyme **II**

Reference crude unconjugated PNA **II**:

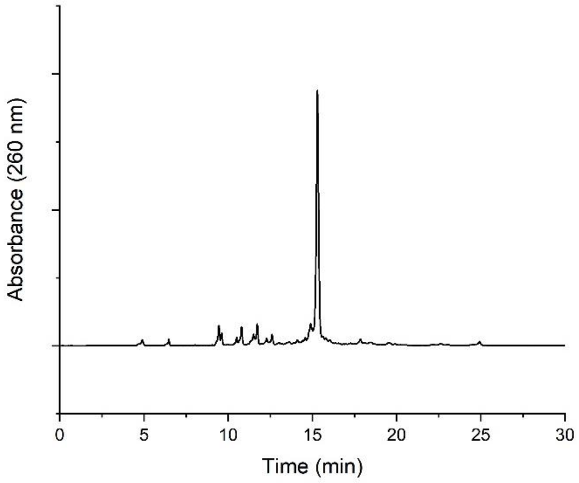

Crude PNAzyme **II** (post conjugation):

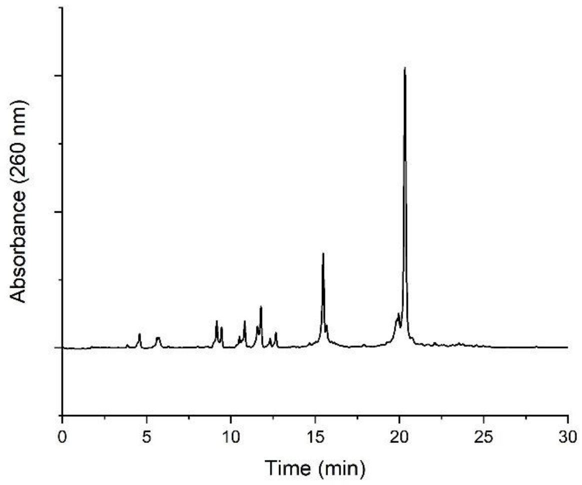

Purified PNAzyme **II**:

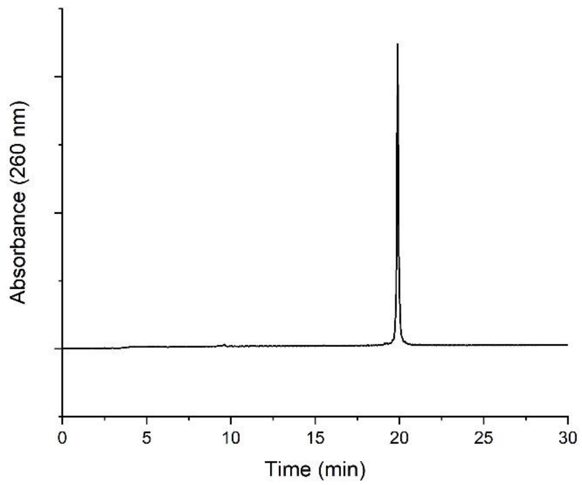

Supplement: Supplementary file 1 [file molecules-24-00672-s001.pdf]
